# Gut microbiota on cardiovascular diseases-a mini review on current evidence

**DOI:** 10.3389/fmicb.2025.1690411

**Published:** 2025-11-06

**Authors:** Shouhong Zhang, Jing Li, Liping Li, Xingxing Yuan

**Affiliations:** 1 Department of Cardiology, Heilongjiang Academy of Traditional Chinese Medicine, Harbin, China; 2 Department of Graduate School, Heilongjiang Academy of Traditional Chinese Medicine, Harbin, China; 3 Department of Gastroenterology, Heilongjiang Academy of Traditional Chinese Medicine, Harbin, China

**Keywords:** gut microbiota, metabolites, cardiovascular disease, biological pathways, therapeutic strategies

## Abstract

The gut microbiome has emerged as a critical modulator of cardiovascular disease (CVD) risk, offering a novel frontier for therapeutic intervention. This mini-review synthesizes current evidence on how probiotic-like bacteria and their metabolites mediate protective physiological mechanisms against CVD. Drawing from both animal models and human clinical trials, we elucidate the biological pathways, including trimethylamine-N-oxide (TMAO), short-chain fatty acids (SCFAs), and bile acid metabolism, through which the gut microbiota influences hypertension, atherosclerosis, and heart failure. Furthermore, we examine microbiota-based strategies such as dietary modification, fecal microbiota transplantation (FMT), and pharmacological agents aimed at restoring microbial homeostasis. Despite promising mechanistic insights, human trials have yet to consistently demonstrate significant clinical benefits in reversing CVD outcomes via gut microbiota modulation. This review underscores the necessity of moving from correlation to causation, highlighting current limitations and future prospects for leveraging gut microbiome research in the development of personalized, effective therapeutic strategies for cardiovascular diseases.

## 1 Introduction

In the pursuit of effective and sustainable medicinal intervention, approaches with minimal adverse effects are highly prioritized. While antibiotics, and endocrine drugs, and metabolic therapies aim to promote health, they often face challenges such as pathogen resistance or detrimental side effects on the host. In recent years, the emerging field of gut microbiota medicine has introduced a range of non-pharmacological interventions, offering promising alternatives (Bajinka et al., 2022, 2023). Notably, macromolecules and metabolites derived from dietary supplements are being extensively studied for their potential to reverse life-threatening conditions, including metabolic disorders, cardiovascular disease (CVD), neurodegenerative diseases, and cancer (Yang and Cong, 2021). Alterations in gut microbiota composition can lead to low-grade, systemic, and local inflammation, directly contributing to the development of metabolic disorders such as obesity, diabetes mellitus, CVD, and microvascular complications (Yang L. et al., 2021; Hasani et al., 2024).

The gut microbiota has emerged as a powerful regulator of host physiology, attracting significant scientific interests in its role in reversing metabolic disorders. It mediates host physiology through various mechanisms, including signaling via receptor ligands, serving as substrates for host enzymes, and providing metabolites as energy sources. Through these interactions, gut microbiota plays a direct role in the pathogenesis of CVD. For instance, the breakdown of dietary nutrients such as choline, coupled with lipid metabolism and host immune system modulation, can lead to the development of atherosclerosis (Dovi et al., 2022). Current strategies to mitigate these conditions focus on reducing trimethylamine (TMA) to trimethylamine-N-oxide (TMAO) using compounds like 3,3-dimethyl-1-butanol and archaebiotics. Additionally, converting high fiber diets into TMA precursors to promote beneficial bacterial populations is being explored as an anti-atherogenic approaches (Anbazhagan et al., 2017).

Beyond metabolic pathways, the gut microbiota also interacts with systemic immunity to influence CVD progression. Studies using murine lupus models have revealed connections between gut microbiota dysbiosis, antiphospholipid syndrome (APS), and autoimmune-driven vascular damage. Altered intestinal IgA levels and molecular mimicry between microbial proteins and host autoantigens may exacerbate thrombosis and endothelial dysfunction, while dietary short-chain fatty acids (SCFAs) could mitigate these effects through immunomodulation (van Mourik et al., 2022). Importantly, the integrity of the gut barrier and microbiota homeostasis is closely associated with systemic inflammation-a key driver of both CVD and cerebrovascular conditions. For example, ischemic stroke shares overlapping risk factors and inflammatory pathways with CVD, suggesting a broader role of gut microbiota in circulatory health (Wei et al., 2021). However, the mechanisms by which gut microbiota directly regulates CVD pathogenesis, particularly through metabolites and endothelial interactions, remain a critical research frontier.

Among CVD-related interventions, dietary phytochemicals such as anthocyanins exemplify the therapeutic potential of microbiota-mediated approaches. Anthocyanins confer antioxidant effects by activating nuclear factor erythroid 2-related factor 2 and suppressing pro-inflammatory cytokines, thereby improving endothelial function and nitric oxide bioavailability (Xin et al., 2024). These findings highlight the bidirectional crosstalk between dietary components, gut microbiota, and cardiovascular homeostasis. Giving the growing evidence supporting the role of gut microbiota in mediating CVD, a systematically analysis of the underlying biological mechanisms is crucial. While human clinical trials provide valuable insights, complementary studies using animal models are essential to elucidate these mechanisms.

To comprehensively map the gut microbiota’s role in CVD, a systematic literature search was conducted in PubMed, Web of Science, and Scopus (2013–2023) using keywords including “gut microbiota,” “cardiovascular disease,” “TMAO,” “SCFAs,” and “inflammation” combined with Boolean operators. Included studies investigated the mechanistic role of gut microbiota in cardiovascular diseases in human or animal models and were published in English. Exclusion criteria covered non-peer-reviewed articles and studies lacking mechanistic insights. Data on study design, findings, and mechanisms were extracted independently by two reviewers, with discrepancies resolved through consensus. Reference lists of relevant articles were also screened.

This mini-review aims to provide a comprehensive overview of recent studies on gut microbiota-mediations interventions for CVD, drawing from both human and animal research over the past decade, sourced from various databases. This review innovatively synthesizes a decade of human and animal studies to map the gut microbiota’s role in CVD through specific metabolites and pathways. It critically evaluates the translational gap between mechanistic insights and clinical applications, highlighting the lack of robust human trial data. By proposing advanced future directions, like CRISPR-based microbiome editing and species-specific FMT, it provides a forward-looking framework for moving from correlation to causation, paving the way for targeted, microbiota-based personalized therapeutics in CVD.

## 2 Gut microbiota-induced risk factors for cardiovascular disease

The risk factors for CVD include hypertension, diabetes, hypercholesterolemia, obesity, chronic inflammation, and genetic factors (Kjeldsen, 2018; Dal Canto et al., 2019; Alfaddagh et al., 2020; Foger et al., 2020; Powell-Wiley et al., 2021; Tada et al., 2022). Among these, gut microbiota diversity, as a key regulator of metabolism and dietary intake, plays a pivotal role in driving the pathological processes of CVD, particularly hypertension, atherosclerosis, and heart failure (Jia et al., 2023).

Hypertension, as a key risk factor for cardio-cerebral vascular diseases, can induce gut microbiota dysbiosis and gut barrier dysfunction. This is mediated by the influx of hydrogen sulfide, lipopolysaccharide (LPS), and pathogenic bacteria, coupled with a reduction in SCFA-producing bacterial populations. These changes lead to increased intestinal permeability and disruption of tight junction proteins (Luqman et al., 2024). Furthermore, reduced alpha diversity and an increased abundance of LPS-producing Gram-negative bacteria contribute to pro-inflammatory responses, resulting in dysregulated blood pressure and hypertension.

Increased gut permeability is strongly associated with LPS translocation into systemic circulation, yet therapeutic targets to mitigate CVD risk remain elusive. Although interventions targeting bile acid receptors show potential in reducing atherosclerosis progression, TMAO has emerged as a pro-atherogenic metabolite, representing one of multiple pathways involved in CVD pathogenesis (Yang et al., 2023). Elevated cholesterol levels are another critical risk factor for CVD. Through gut microbiota modulation, probiotics from the genera *Bifidobacterium* and *Lactobacillus* have demonstrated efficacy in controlling cholesterol levels in clinical studies (Fuentes et al., 2013; Costabile et al., 2017; Marras et al., 2021). Moreover, next-generation probiotics, such as *Bacteroides* spp., and *Akkermansia* show promise in specifically lowering cholesterol levels (Verhaar et al., 2020). Gut microbiota-derived metabolites, include TMAO, SCFAs, polyphenols, and bile acids, are essential for maintaining the healthy function of cardiovascular organs (Massey et al., 2022).

## 3 Gut microbiota-induced mechanisms for cardiovascular disease

Distinct gut dysbiosis profiles are closely associated with specific CVD subtypes, reflecting the complex interplay between microbiota composition and disease pathophysiology (Datta et al., 2024). In metabolic disorders such as T2DM, obesity, and hyperlipidemia, the gut microbiota is characterized by a significant reduction in the abundance of *Akkermansia* and *Bifidobacterium*, two genera known for their roles in maintaining metabolic homeostasis and gut barrier integrity. In contrast, atherosclerosis is associated with an elevated Firmicutes/Bacteroidetes ratio, a microbial signature often linked to pro-inflammatory states and impaired lipid metabolism. Furthermore, coronary heart disease shows a unique microbial profile marked by increased *Collinsella* abundance, a genus implicated in promoting inflammation and cholesterol accumulation. Meanwhile, myocardial infarction exhibits a transient microbial shift, featuring enrichment of *Lactobacillus* and depletion of *Bacillus*, which may reflect acute inflammatory responses and oxidative stress during the ischemic event.

From a metabolic perspective, gut microbiota-derived metabolites play pivotal roles in CVD pathogenesis through multiple mechanisms. Elevated TMAO levels, generated from dietary choline via gut microbial metabolism and hepatic flavin monooxygenase-3 conversion, are associated with CVD risk factors including obesity, type 2 diabetes mellitus (T2DM), hyperlipidemia, as well as specific CVD manifestations such as coronary heart disease and myocardial infarction by promoting platelet hyperreactivity, atherosclerosis, and heart failure (Zhu et al., 2016; Guasti et al., 2021; Zhang et al., 2021). The gut microbiota also influences bile acid metabolism, which activates nuclear receptors such as the farnesoid X receptor (FXR) and pregnane X receptor (PXR). These receptors regulate lipid homeostasis by downregulating triglyceride levels and attenuating pro-inflammatory NF-κB signaling, thereby modulating atherosclerosis progression and coronary heart disease (Wu et al., 2021). Conversely, dysbiosis-driven metabolites like phenylacetylglutamine exacerbate thrombosis and in-stent stenosis (Chassaing et al., 2015; Lahnwong et al., 2018), while hydrogen sulfide exerts protective effects by inhibiting nucleotide-binding oligomerization domain-like receptor protein 3 (NLRP3) inflammasome activation via purinoreceptor-7 blockade (Jia et al., 2020). Indole-3-propionic acid (IPA), a microbial metabolite derived from dietary tryptophan by gut microbiota such as *Lactobacillus reuteri*, *Clostridium caloritolerans*, *Clostridium sporogenes*, and *Peptostreptococcus*. IPA enhances gut–blood barrier function through the expression of tight junction proteins and claudins, activates the aryl hydrocarbon receptor, and protects against lipid peroxidation and oxidative damage (Konopelski and Mogilnicka, 2022). Furthermore, IPA stimulates macrophage reverse cholesterol transport by upregulating ABCA1 expression (Schwartz et al., 2013). As gut microbiota translocates to the aortic artery due to defect in gut barrier integrity, microbial-derived metabolites and inflammation results in renal insufficiency and inflammation-signaling pathway. While gut microbiota modulation of drug efficacy against CVD is harnessed, its combination with physical exercise will lead to healthier cardiovascular organ functions (Jia et al., 2019).

## 4 Gut microbiota-mediated therapeutic strategies for cardiovascular disease

To prevent CVD, current strategies include evidence-based approaches such as dietary and lifestyle modifications, pharmacological interventions, as well as novel approaches like fecal microbiota transplantation (FMT) and NLRP3 inflammasome inhibition. Mechanistic studies have shown that these interventions may contribute to reduced risks of heart failure and atherosclerosis, though clinical evidence varies across different modalities. Regular physical exercise and supplementation with probiotics, prebiotics, or their combination as synbiotics have been shown to ameliorate cardiac hypertrophy and fibrosis. Furthermore, adopting healthy lifestyle practices enhances microbiome composition and function, thereby augmenting the production of beneficial metabolites essential for gastrointestinal tract homeostasis (Zhao and Wang, 2020; Usman et al., 2024). Through gut microbiota-artery axis, dietary components such as omega-3 polyunsaturated fatty acids, sphingomyelin, and phosphatidylcholine play pivotal roles in lipid metabolism and the progression of atherosclerosis (Shi et al., 2024). Probiotics, in particular, stabilize the gastrointestinal tract dynamics, generating metabolites with cardioprotective properties, thus advancing precision medicine for cardiometabolic complications. Conversely, high-salt dietary supplements reduce the abundance of *Lactobacillus*, leading to an increase in T helper 17 (Th17) cells, which are biomarkers for salt-sensitive hypertension. The Mediterranean diet, characterized by reduced saturated fatty acids, phosphate, and sodium, along with enriched nitrate, fiber, and antioxidants, promotes gut microbiota diversity. This ultimately mitigates oxidative stress while enhancing antioxidant functions and nitric oxide bioavailability, thereby improving cardiac and vascular function (Barrea et al., 2019).

In addition to dietary and lifestyle interventions, the introduction of healthy gastrointestinal tract microbiome to patient’s FMT is effective for controlling *Clostridium difficile*. Emerging evidence suggests its potential in improving insulin sensitivity and plasma triglyceride levels, thereby enhancing cardiometabolic health (Miranda et al., 2018). This is evidenced by a downregulation of myocarditis incidence and an increase in Bacteroidetes abundance. Pharmacological interventions targeting gut microbiota composition and dynamics are crucial for attenuating CVD-related dysbiosis. Dipeptidyl peptidase-4 (DPP-4) inhibitors mitigate cardiovascular risks by attenuating high-fat-diet-induced dysbiosis, promoting the abundance of SCFAs-producing microbiota, and reducing the Bacteroidetes to Firmicutes ratio (Zhang et al., 2023). Biguanides, such as metformin, reduce the risk of myocardial infarction in patients with T2DM by regulating the incretin pathway and enhancing peripheral glucose uptake. This is achieved through increased pancreatic and plasma levels of glucagon-like peptide-1, a precursor for SCFAs, which exert cardioprotective effects (Bu et al., 2022). Additionally, metformin downregulates bile acids absorption by inhibiting the apical sodium-dependent bile acid transporter, thereby interfering with primary bile acids-mediated FXR activation. This process is facilitated by SCFA-producing genera such as *Bifidobacterium bifidum*, *Butyrivibrio*, and *Megasphera*. Furthermore, α-glucosidase inhibitors, such as acarbose, upregulate bile salt hydrolase activity, promoting beneficial genera like *Bifidobacterium* and *Lactobacillus* while suppressing pathogenic genera such as *Clostridium*, *Alistipes*, and *Bacteroides*, thereby contributing to cardiometabolic homeostasis (Montandon and Jornayvaz, 2017; Hu et al., 2019).

Targeting the NLRP3 inflammasome, a sensor of deleterious endogenous and exogenous stimuli, is a promising therapeutic strategy for mitigating pro-inflammatory signaling and CVD progression, particularly atherosclerosis. Cholesterol crystals activate the NLRP3 inflammasome, increasing the risk of atherosclerotic plaque formation (Morgan et al., 2014). TMAO, a key trigger of NLRP3 inflammasome activation, induces endothelial barrier dysfunction and hyperpermeability, ultimately leading to cardiac fibrosis. Therefore, interventions targeting NLRP3 inflammasome activation are critical for attenuating atherosclerosis. Statins, analogs of HMG-CoA reductase inhibitors, mitigate oxidative stress, endothelial dysfunction, cardiomyocyte apoptosis, and cardiac hypertrophy (Ning et al., 2010; Viollet et al., 2012). Statins, including rosuvastatin, impede the TLR4/MyD88/NF-κB signaling pathway, a key activator of NLRP3 inflammasome, and are effective in treating diabetic cardiomyopathy (Duewell et al., 2010). Simvastatin enhances endothelial barrier integrity by upregulating tight junction protein expression, reducing vascular endothelial hyperpermeability, and mitigating hyperglycemia-associated endothelial dysfunction (Rajamaki et al., 2010). Mechanistically, simvastatin inhibits HMGB1 release in aortic endothelial cells, thereby obstructing NLRP3 inflammasome activation. Additionally, the natural NLRP3 inflammasome inhibitor, arglabin, exhibits anti-atherogenic effects in high-fat-diet-induced murine models (Zhang et al., 2022).

**FIGURE 1:**
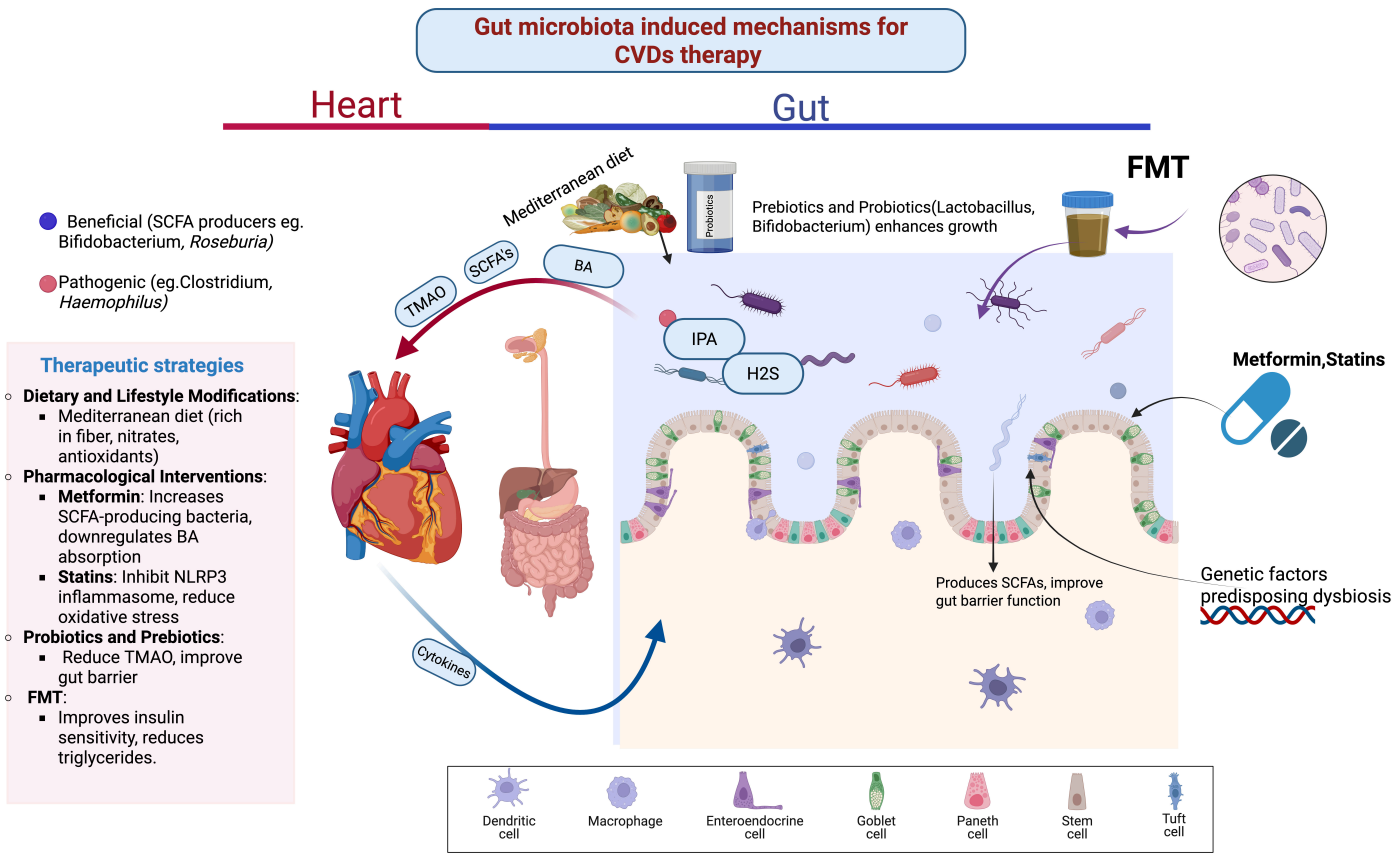
Gut microbiota induced mechanisms for cardiovascular disease therapy.

Acetate-producing bacteria reduce blood pressure and alleviate cardiac fibrosis and hypertrophy, while probiotics modulate oxidative stress, inflammation, renin-angiotensin system overactivity, vascular resistance, and hyperlipidemia. *Lactobacilli* increase SCFA production and reduce toxin levels, thereby inhibiting atherosclerosis progression. TMAO levels can be reduced by inhibiting TMA production in the gastrointestinal tract, and flavonoids inhibit TMA lyase activity, offering therapeutic potential for coronary heart disease. Oat fiber prevents atherosclerosis by blocking the TLR4 signaling pathway, modifying lipid metabolism, and reducing NF-κB p65 expression (Gao et al., 2022). Furthermore, intestinal mucosal barrier integrity is preserved through alterations in metabolites such as 1-methylguanosine, 2-methylguanosine, isobutyrylcarnitine, and valerylcarnitine. Fish oil-derived long-chain monounsaturated fatty acids (LCMUFAs) have demonstrated cardiovascular risk reduction in murine models, with reduced TMAO levels and improved endothelial function (Tsutsumi et al., 2021). LCMUFAs also increase *Akkermansia* abundance while reducing Firmicutes and Bacteroidetes, thereby balancing the intestinal microenvironment through SCFA regulation and glucagon-like peptide modulation. Additionally, LCMUFAs attenuate atherosclerosis by reducing macrophage infiltration, regulating inflammation, and lowering serum levels of branched-chain amino acids (Yang et al., 2016; Figure 1 and Table 1).

**TABLE 1:** Human clinical trials on gut microbiota mediation to cardiovascular effects.

Subject	Main findings	Conclusion	References
• Antidiabetic and hypolipidemic regimen on postprandial lipidemia in T2DM	• Alterations in fecal *Bifidobacterium breve* levels. • Four fadD genes encoding long-chain Acyl-CoA synthetase were identified. • Decreased the concentration of free fatty acid.	• Berberine and *Bifidobacterium breve* exert a synergistic hypolipidemic effect on postprandial lipidemia, improving lipid profiles and reducing CVD risk in T2DM patients.	Wang et al., 2022
• Vegetarian diet on cardiovascular risk in ischemic heart disease	• Decreased levels of ○ Oxidized low-density lipoprotein cholesterol ○ Total cholesterol ○ Low-density lipoprotein cholesterol ○ Body weight • Shifts abundance of ○ Ruminococcaceae ○ Lachnospiraceae ○ Akkermansiaceae • Changes in ○ L-carnitine ○ Acylcarnitine metabolites ○ Phospholipids	• A vegetarian diet combined with optimal medical therapy reduces oxidized low-density lipoprotein cholesterol levels, improving cardiometabolic health.	Djekic et al., 2020
• Weight-loss via energy-reduced MedDiet and physical activity	• Greater weight loss and improved symptoms of cardiovascular symptoms • Shifts in ○ Bile acids ○ Ceramides ○ Sphingosines ○ Fatty acids ○ Carnitines ○ Nucleotides ○ Purine metabolites ○ Krebs cycle intermediates • Reduced abundance of ○ *Eubacterium hallii* ○ *Dorea* • Increased alpha diversity	• Energy-reduced MedDiet combined with physical activity reduces cardiometabolic risk factors	Garcia-Gavilan et al., 2024
• Oral rifaximin on cardiovascular toxins and proinflammatory cytokines in chronic kidney disease	• No significant changes in serum TMAO, p-cresol sulfate, indoxyl sulfate, kynurenic acid, deoxycholic acid, or inflammatory cytokines • Differences in colonic bacterial communities and diversity	• Short-term rifaximin treatment failed to reduce gut-derived cardiovascular toxins and in patients with chronic kidney disease.	Kimber et al., 2020
• MedDiet interventions on bile acids and cardiometabolic risk	• Lithocholic acid and ursodeoxycholic acid associated with BMI and serum lipid profiles • Baseline fecal bile acid levels modified BMI reduction, with lower 12-dehydrocholic acid levels	• A novel predictive biomarker for cardiometabolic risk for personalized dietary interventions	Gao P. et al., 2024
• Gut bacteriophage community dynamics after FMT in metabolic syndrome	• Alterations to the gut bacteriophage community	• Bacteriophage dynamics may explain the observed differences in metabolic outcomes post-FMT.	Manrique et al., 2021
• Dietary fibers on cardiometabolic profile	• Decreased *Bacteroides vulgatus* • Increased abundance of ○ *Parabacteroides distasonis* ○ *Fusicatenibacter saccharivorans* ○ An unclassified ○ Acutalibacteraceae ○ *Eisenbergiella* • Decreased levels of ○ Total cholesterol ○ LDL cholesterol ○ Insulin ○ HOMA	• Dietary fibers as nutritional tool can improve cardiometabolic profile.	Ranaivo et al., 2022
• Multifunctional nutritional strategy on cardiometabolic diseases	• Reduced ○ Intestinal inflammation ○ Fecal calprotectin ○ Endotoxemia • No alteration in systemic inflammation • Decreased serum branched-chain amino acids • Increased abundance of ○ *B. ovatus* ○ *B. uniformis* ○ Butyriciproducens • Unclassified Christensenellaceae.CAG-74	• A strategy to target low-grade inflammation via multi-target approach	Hornero-Ramirez et al., 2025
• Omega-3 or inulin supplementation on cardiovascular effects	• Inulin supplementation increases abundance of ○ *Bifidobacterium* ○ Lachnospiraceae • Omega-3 supplementation increases abundance of ○ *Coprococcus* spp. ○ *Bacteroides* spp. • Omega-3 significant decreases ○ Fatty-liver associated *Collinsella* spp. ○ Inulin increases ○ Butyrate ○ Iso-valerate ○ Iso-butyrate ○ Nearly increases butyrate	• Dietary omega-3 alters gut microbiota composition and some cardiovascular effects	Vijay et al., 2021
• Gut-cardiovascular axis in low-resource individuals	• Shared microbiota-blood pressure relationships between dyads • Host-microbiota responses to lifestyle interventions	• The gut-cardiovascular axis is a potential target for reducing future CVD risk in low resource populations.	Hill et al., 2022
• Choline utilization C/D (cutC/D) gene and thrombosis	• Microbial cutC-dependent TMA/TMAO production is crucial for transmitting increased thrombosis and platelet reactivity	• Microbial choline TMA-lyase pathway is a molecular target for atherothrombotic heart disease treatment	Skye et al., 2018
• Small metabolites with hypertension risk	• A shift in baseline levels of 26 metabolites owing to hypertension • Amino acids are negatively associated with risk of hypertension • The essential amino acids are threonine and phenylalanine • Increased lyxose has higher risk of hypertension	• Low amino acid levels and gut microbiota play an important role in hypertension pathogenesis	Hao et al., 2016

## 5 Nutrition, the microbiome, and cardiovascular disease pathogenesis

This section highlights a complex, bidirectional relationship between diet, gut microbiota composition, and host cardiovascular health. The molecular mechanisms underpinning this relationship involve microbial metabolite production, systemic inflammation, oxidative stress, and endothelial function.

### 5.1 Oxidative stress and microbial metabolites

Oxidative stress is a critical molecular mechanism through which gut dysbiosis contributes to CVD. An imbalanced microbiome can produce metabolites that either exacerbate or ameliorate oxidative damage. Certain dietary patterns foster bacteria that produce metabolites with potent antioxidant properties. For instance, the high-fiber rye intervention in the RyeWeight study led to increased plasma levels of microbial metabolites like indolepropionic acid and enterolactone (Wang et al., 2024). These compounds are known for their antioxidant activities, which can mitigate oxidative damage to lipids and proteins in the vascular endothelium, a key step in atherogenesis. Similarly, the hydroxytyrosol (HT) study demonstrated that responders to this phenolic compound exhibited improved glutathione metabolism, a master regulator of the cellular antioxidant defense system (Li et al., 2025). This suggests that HT’s benefits may be mediated through both direct antioxidant effects and the modulation of microbial communities that support endogenous antioxidant pathways. Direct evidence comes from the trial on postbiotic supplementation in stroke patients, which showed a significant reduction in the oxidative stress marker malondialdehyde (MDA) and an increase in total antioxidant capacity (TAC) (Chavez-Alfaro et al., 2025). This indicates that postbiotics (inanimate microorganisms and/or their components) can confer systemic antioxidant benefits, likely by reducing the production of pro-oxidant molecules by the host’s immune system or by the microbiota itself.

### 5.2 Inflammation and endothelial dysfunction

Systemic inflammation is a cornerstone of CVD pathogenesis, and the gut microbiome is a primary regulator of this process. Diet directly influences the inflammatory tone via microbial signaling. Dysbiosis can increase gut permeability (“leaky gut”), allowing the translocation of bacterial fragments like LPS into the bloodstream. LPS is a potent endotoxin that triggers systemic inflammation by activating immune cells and promoting the release of pro-inflammatory cytokines such as IL-1β, IL-6, and TNF-α (Fan et al., 2025). This chronic, low-grade inflammation damages the endothelium and promotes atherosclerosis.

The abstracts show that dietary interventions can reverse this. The CADIMED trial (Ng et al., 2025) and the cycling diet study aim to or demonstrate a reduction in CVD risk factors by promoting anti-inflammatory microbial patterns (Verhaar et al., 2024). The casein peptide study explicitly links its antihypertensive effect to anti-inflammatory and antioxidant effects that improve endothelial function (Gawlik-Kotelnicka et al., 2024). Furthermore, the postbiotic trial confirmed a direct reduction in IL-1β and hs-CRP following intervention, underscoring the gut-inflammatory axis (Spasova et al., 2024).

Butyrate, a SCFA produced by bacterial fermentation of dietary fiber, is generally considered anti-inflammatory. However, the trial by Verhaar et al. (2024) presents a surprising finding: oral butyrate supplementation increased blood pressure in hypertensive patients (San Diego et al., 2025). This critical result indicates that the molecular context (route of administration, baseline health, concomitant diet) drastically alters the effect of a single metabolite. It warns against simplistic supplementation and highlights the need for a holistic, diet-based approach to modulate the entire microbial ecosystem for balanced SCFA production. Conversely, the study by San Diego et al. (2025) found that the blood pressure response to a butyrate enema was correlated with intake of specific food groups (vegetables, whole grains), not just the nutrient itself, emphasizing the importance of the dietary matrix (Rahimi et al., 2024).

### 5.3 Pro-atherogenic microbial metabolites

The gut microbiome can generate metabolites that directly contribute to CVD pathology. The trial by Spasova et al. (2024) focuses on TMAO, a well-established independent risk marker for CVD (Yang et al., 2025). Gut bacteria metabolize dietary nutrients like L-carnitine (red meat) and choline (eggs, liver) into TMA, which is then oxidized in the liver to TMAO. TMAO promotes atherosclerosis by stimulating foam cell formation, enhancing platelet reactivity, and inducing endothelial dysfunction. Interventions aimed at reducing TMAO-producing bacteria or their activity are a direct molecular strategy for CVD risk reduction.

### 5.4 Impact of specific nutrients and dietary fibers on microbial ecology

The molecular effects are ultimately driven by diet-induced shifts in the microbial population. Increased fiber intake, as seen with defatted rice bran bread and the high-fiber rye diet, promotes the growth of beneficial bacteria like *Faecalibacterium prausnitzii* (a major butyrate producer) and *Bifidobacterium* (Pontes et al., 2025; Union Caballero et al., 2025). These shifts are associated with improved metabolic outcomes, such as increased HDL cholesterol. The physical therapy study also showed that a diet enriched with dietary fibers increased fecal levels of propionic and butyric acid, which was correlated with reduced systemic inflammation and improved vascular stiffness (Noguera-Navarro et al., 2025). The study by Wang et al. (2024) reveals that the molecular and microbial environment is dynamic. Repeatedly adopting and abandoning a healthy dietary pattern led to a cycling pattern in microbial taxa (*Collinsella*, *Mediterraneibacter*) that was mirrored by cycling in LDL-C and total cholesterol. This demonstrates that the molecular benefits of a healthy diet are transient and dependent on sustained dietary habits, as the microbiome rapidly reverts to its baseline state.

### 5.5 Therapeutic targeting

Multi-strain probiotics improved glucose homeostasis (HbA1c) in hypertensive individuals (Noguera-Navarro et al., 2025). More strikingly, probiotic supplementation prior to cardiopulmonary bypass surgery significantly reduced the incidence of acute gastrointestinal injury, likely by preserving gut barrier integrity and preventing bacterial translocation and subsequent systemic inflammation and oxidative stress (Petelina et al., 2025). The study by Gawlik-Kotelnicka et al. (2024) further suggests probiotic efficacy may be enhanced in individuals with a “leaky gut” or specific immunometabolic profiles (Li-Hua and Bajinka, 2025). The FMT trial for hypertension provided proof-of-concept that altering the gut microbiome can affect blood pressure (Bajinka et al., 2025). Although the effect was unsustainable, the study identified specific bacteria (*Eggerthella lenta*, *Erysipelatoclostridium ramosum*) whose decreased abundance was correlated with reduced BP, and others whose increase was beneficial. It also linked these microbial shifts to changes in blood pressure-modulating metabolites like tyrosine, glutamine, and phenylalanine, outlining a clear molecular pathway from microbiota to host physiology.

The molecular pathogenesis of heart diseases is profoundly influenced by the gut microbiome, which acts as a key interpreter of nutritional intake. Diets rich in fiber and polyphenols (Mediterranean, high-fiber rye) promote a microbial ecosystem that produces beneficial metabolites (SCFAs, antioxidants), reduces inflammation, and protects the endothelium. Conversely, poor dietary patterns lead to dysbiosis, characterized by increased production of pro-inflammatory molecules, pro-oxidant species, and pro-atherogenic metabolites like TMAO, thereby driving CVD progression. The findings caution against the isolated use of microbial metabolites such as butyrate pills and instead advocate for a whole-diet approach to sustainably modulate the complex molecular dialogue between nutrition, the microbiome, and the host cardiovascular system.

## 6 Limitations to gut microbiota-mediating cardiovascular disease

Despite advancements in targeted preventive and personalized approaches, including FMT, dietary modulation, and pharmacological interventions, significant limitations persist in gut microbiota-mediation strategies for delaying the onset and progression of CVD episodes. A primary challenge lies in the incomplete understanding of the biological mechanisms underlying the pathophysiological state induced by gut microbiota dysbiosis, which contributes to CVD development. While metformin demonstrates effective cardioprotective benefits in diabetic patients, its therapeutic effects in non-diabetic individuals remain mechanistically unexplained. Furthermore, although preliminary evidence suggests improvement in ischemic risk vascular function among angina patients, large-scale randomized controlled trials with extended follow-up periods are required to establish robust clinical significance (Jadhav et al., 2006; Holman et al., 2017; Luo et al., 2019). Notably, metformin intervention has shown no significant impact on carotid intima-media thickness, a well-established CVD biomarker (Chen Y. et al., 2020).

Additional limitations in gut microbiota-mediated CVD pathophysiology include the inconsistent cardioprotective effects of α-glucosidase inhibitors. Specifically, coronary artery disease patients with impaired glucose tolerance demonstrate no significant cardiometabolic improvement following acarbose administration. Moreover, sodium glucose co-transporter 2 inhibitors, despite their demonstrated effects in reducing cellular apoptosis, mitochondrial dysfunction, and cardiovascular inflammation, exhibit no discernible impact on gut microbiota homeostasis (Zeng et al., 2021). The therapeutic application of FMT faces challenges related to donor-recipient incompatibility, particularly concerning extra-intestinal complications such as infections and endotoxin-related adverse events, necessitating rigorous safety evaluations (Cao et al., 2025).

The role of free sulfate concentrations in mitochondrial complex IV function remains unclear, potentially leading to impaired oxygen consumption and reduce butyrate oxidation in the colon following non-digestible carbohydrates administration (Rastall et al., 2022). These prebiotics possess emulsification property that may alter gut microbiota composition and potentially facilitate epithelium bacterial translocation, increasing the risk of septicemia (Allam-Ndoul et al., 2020). While gut microbiota modulation for CVD prevention and treatment is gaining increasing attention, the imperative for well-designed studies adhering to stringent clinical safety and efficacy standards remains paramount.

The field must progress from establishing correlations to demonstrating causality in gut microbiota-mediated CVD mechanisms. This requires the identification of functional metabolites associated with specific molecular pathways and the characterization of microbial species and strains producing bioactive compounds with distinct cardiac phenotypes. Furthermore, the standardization of experimental conditions and optimization of FMT protocols, from sample collection to storage, remain areas requiring resolution due to ongoing procedural controversies.

## 7 Prospects in gut microbiota-mediated cardiovascular disease

The integration of advanced technologies, such as CRISPR/Cas9, enables precise modulation of gut microbiota metabolic pathways, thereby enhancing the targeted expression of cardioprotective metabolites. This approach will facilitate the identification of specific roles played by gut microbiota-derived metabolites in attenuating CVD pathogenesis, while elucidating the underlying biochemical processes. In addition, to comprehensively understand gut microbiota-derived metabolic changes in CVD, human metabolomics studies should be integrated with bacterial proteomic and metagenomics, providing a holistic view of the onset and progression of CVD episodes. Moreover, FMT strategies focusing on species-specific interventions rather than phyla or genera, along with the use of cytostatic agents instead of cytotoxic drugs, will ensure both target specificity and the maintenance of gut microbiota homeostasis (Ahmad et al., 2024).

Cardiovascular disease-associated conditions, such as hypertension, coronary atherosclerosis and heart failure, represent a significant global health burden, contributing to elevated mortality rates. The bidirectional interplay between these conditions and gut microbiota is mediated by well-defined biological processes, highlighting the functional role of gut microbiota in cardiovascular health. Given that arteriosclerosis is often diagnosed at advanced stages, characterized by elevated blood glucose, lipid, and insulin levels, as well as persistent low-grade inflammation and insulin resistance, there is an urgent need for microbiota-targeted individualized strategies for both the prevention and management of CVD (Hsu et al., 2019). Reduced diversity of SCFAs producing bacteria and the proliferation of pathogenic bacteria in the gut microbiota can lead to increased levels of TMAO, a known risk factor for CVD. Supplementation with probiotics and prebiotics can promote the dominance of beneficial bacterial populations, thereby inhibiting the conversion of dietary lecithin, L-carnitine, and choline into TMA, which is subsequently oxidized to TMAO in the liver. Notably, TMAO serves as a biomarker for stroke, myocardial infarction, and mortality, while SCFAs exert systemic anti-inflammatory effects, serving as energy sources for colonocytes and regulators of blood pressure. Maintaining a consistent supply of SCFAs is crucial for delaying the onset of hypertension, and bile acid biosynthesis, mediated by receptors such as takeda G protein-coupled receptor 5, PXR, and FXR, is significantly influenced by gut microbiota in CVD (Martins et al., 2024; Ronen et al., 2024).

In summary, the three primary gut microbiota-derived metabolites such as SCFAs, TMAO, and bile acids are promising biomarkers for CVD. Their targeted modulation offers novel therapeutic strategies for CVD treatment. Future therapeutic approaches will focus on harnessing molecular mechanistic studies to elucidate the bidirectional biological pathways connecting gut microbiota and CVD risk. Investigating specific microbial taxa, including non-bacterial microorganisms, as well as the shifts in metabolite profiles and their associated metabolic diseases, will provide deeper insights into gut microbiota-mediated prevention and management of CVD.

Hypercholesterolemia, a well-established risk factor for CVD, can be mitigated through gut microbiota modulation via supplementation with prebiotics, probiotics, and statins. These interventions regulate cholesterol homeostasis through metabolites such as TMAO, SCFAs, and bile acids (Vourakis et al., 2021). A meta-omics perspective offers profound implications for understanding the role of gut microbiota in CVD (Xu and Yang, 2021). Beyond dietary interventions, the detection of biomarkers represents another promising strategy for reducing CVD risk (Evans et al., 2020). The interactions between phytochemicals and gut microbiota-mediated histone acetylation, adipose tissue dysfunction, blood pressure regulation, and other bioactive compounds are areas of growing interest (Pieczynska et al., 2020; Yang X. et al., 2021; Yan et al., 2022; Jiang et al., 2024). Additionally, the role of gut microbiota and its metabolites in reverse cholesterol transport, stroke-related risk factors, and exercise-mediated protection in atherosclerotic cardiovascular disease warrants further investigation (Gao C. et al., 2024; Zhong et al., 2024).

With far-reaching clinical applications, gut microbiota research has been significantly advanced through the use of cutting-edge technologies, enabling robust and sensitive analyses. Although mechanistic studies remain incomplete, biochemical and molecular methodologies are yielding increasingly reliable results in this field. The detection of biomarkers via metabolomics analysis holds promise for personalized medicine, offering alternative adjunct therapies through gut microbiota manipulation (Chen L. et al., 2020). Despite clear evidence of the gut-immune-B2 cell axis, including B cell-mediated humoral immunity via TLR signaling pathways, the modulation of atherosclerosis-associated immune responses requires further scientific validation (Forkosh and Ilan, 2019). The heart-gut axis has emerged as a novel therapeutic target for congestive heart failure and atherosclerosis (Singh et al., 2024).

From a clinical perspective, T2DM patients at risk of developing CVD exhibit synergistic hypolipidemic effects following postprandial lipidemia reduction induced by berberine and *Bifidobacterium breve*. The dynamics of the gut bacteriophage community post-FMT are critical for metabolic syndrome subjects. Vitamin D supplementation has been shown to reduce oxidized low-density lipoprotein cholesterol levels, thereby improving cardiometabolic health (Ostadmohammadi et al., 2019). Weight loss interventions based on energy-reduced Mediterranean diets have demonstrated significant improvements in CVD risk factors. Mediterranean diets interventions targeting bile acids represent a novel approach to biomarker production for cardiometabolic risk, as do dietary fibers in improving cardiometabolic profiles. Microbiota-focused strategies, such as omega-3 fatty acids, inulin, and choline utilization for thrombosis-related genes, can enhance cardiometabolic effects. However, short-term rifaximin treatment has failed to reduce gut-derived cardiovascular toxins in patients with chronic kidney disease.

## 8 Conclusion

This mini-review underscores the pivotal role of gut microbiota-derived metabolites, particularly TMAO, SCFAs, and bile acids as both biomarkers and mediators in CVD. TMAO is strongly linked to atherosclerosis and thrombosis, while SCFAs confer anti-inflammatory and blood pressure-regulating benefits. Bile acid metabolism, modulated by receptors such as TGR5, FXR, and PXR, further influences lipid homeostasis and vascular health. Therapeutic strategies like FMT, the Mediterranean diet, and supplementation with omega-3 fatty acids or inulin show promise in reshaping microbial communities and reducing CVD risk. However, translating these interventions into sustained clinical benefits remains challenging. Future efforts must focus on elucidating causal mechanisms, standardizing protocols, and advancing personalized microbiota-targeted therapies. Ultimately, harnessing the gut-heart axis offers a transformative approach for the prevention and treatment of cardiovascular diseases.
